# Google Glass-Directed Monitoring and Control of Microfluidic Biosensors and Actuators

**DOI:** 10.1038/srep22237

**Published:** 2016-03-01

**Authors:** Yu Shrike Zhang, Fabio Busignani, João Ribas, Julio Aleman, Talles Nascimento Rodrigues, Seyed Ali Mousavi Shaegh, Solange Massa, Camilla Baj Rossi, Irene Taurino, Su-Ryon Shin, Giovanni Calzone, Givan Mark Amaratunga, Douglas Leon Chambers, Saman Jabari, Yuxi Niu, Vijayan Manoharan, Mehmet Remzi Dokmeci, Sandro Carrara, Danilo Demarchi, Ali Khademhosseini

**Affiliations:** 1 Biomaterials Innovation Research Center, Division of Biomedical Engineering, Department of Medicine, Brigham and Women’s Hospital, Harvard Medical School, Cambridge, MA 02139, USA; 2 Harvard-MIT Division of Health Sciences and Technology, Massachusetts Institute of Technology, Cambridge, MA 02139, USA; 3 Wyss Institute for Biologically Inspired Engineering, Harvard University, Cambridge, MA 02139, USA; 4 Department of Electronics and Telecommunications, Politecnico di Torino, 10129 Torino, Italy; 5 Doctoral Programme in Experimental Biology and Biomedicine, Center for Neuroscience and Cell Biology, Institute for Interdisciplinary Research, University of Coimbra, 3030-789 Coimbra, Portugal; 6 Biocant — Biotechnology Innovation Center, 3060-197 Cantanhede, Portugal; 7 Federal University of São Francisco Valley, Centro, Petrolina, PE 56304-917, Brazil; 8 Programa de Doctorado en Biomedicina, Universidad de los Andes, Santiago 7620001, Chile; 9 Integrated Systems Laboratory, École Polytechnique Fédérale de Lausanne, 1015 Lausanne, Switzerland; 10 Department of Biomedical Engineering, Polytechnic University of Turin, Turin, Italy; 11 University of Pittsburgh, Pittsburgh, PA 15260, USA; 12 Department of Electrical Engineering and Computer Science, Massachusetts Institute of Technology, Cambridge, MA 02139, USA; 13 Hammond High School, Columbia, SC 29209, USA; 14 College of Life Sciences, Zhejiang University, Hangzhou 310027, China; 15 Department of Bioindustrial Technologies, College of Animal Bioscience and Technology, Konkuk University, Hwayang-dong, Gwangjin-gu, Seoul 143-701, Republic of Korea; 16 Department of Physics, King Abdulaziz University, Jeddah 21569, Saudi Arabia

## Abstract

Google Glass is a recently designed wearable device capable of displaying information in a smartphone-like hands-free format by wireless communication. The Glass also provides convenient control over remote devices, primarily enabled by voice recognition commands. These unique features of the Google Glass make it useful for medical and biomedical applications where hands-free experiences are strongly preferred. Here, we report for the first time, an integral set of hardware, firmware, software, and Glassware that enabled wireless transmission of sensor data onto the Google Glass for on-demand data visualization and real-time analysis. Additionally, the platform allowed the user to control outputs entered through the Glass, therefore achieving bi-directional Glass-device interfacing. Using this versatile platform, we demonstrated its capability in monitoring physical and physiological parameters such as temperature, pH, and morphology of liver- and heart-on-chips. Furthermore, we showed the capability to remotely introduce pharmaceutical compounds into a microfluidic human primary liver bioreactor at desired time points while monitoring their effects through the Glass. We believe that such an innovative platform, along with its concept, has set up a premise in wearable monitoring and controlling technology for a wide variety of applications in biomedicine.

Accurate monitoring and control are key aspects in generating and collecting biological data, from morphology to physiology and respective responses to stimuli. Advancements in the past decades have led to the development of a variety of high-precision biosensors and actuators for unprecedented biomedical applications^1^,^2^,^3^,^4^,^5^. Particularly, the recent advancements in combining them with microfluidic devices have garnered tremendous attention due to their capability of low-volume analysis, high-throughput fluid handling, and miniaturization^6^,^7^,^8^. For example, microfluidic bioreactors have been fabricated to mimic the human physiological system, termed as organs-on-a-chip platforms^9^,^10^,^11^,^12^,^13^,^14^,^15^,^16^,^17^,^18^,^19^,^20^,^21^,^22^. These platforms predict physiological responses with high accuracy, and typically encompass a set of finely designed microfluidic organoid modules interconnected together^10^,^23^, pneumatic-driven valves,^2^,^3^,^4^,^5^ and biosensing units^24^,^25^. However, they generally require frequent, on-demand monitoring and control, since the observation period can last from hours to weeks^9^,^24^,^26^.

The use of conventional desktop computers limits the user mobility and data accessibility, slowing down data-driven decision-making processes. This is especially problematic when monitoring over extended periods of time. Recent advances in mobile technologies such as smartphones and tablets^27^,^28^,^29^,^30^,^31^,^32^,^33^,^34^,^35^,^36^,^37^,^38^, and especially new wearable devices such as bands and smart watches^39^,^40^,^41^, have paved an entirely new avenue for fulfilling these tasks in a much more flexible and remote manner, greatly reducing the labor effort and improving data accessibility. Among all, the Google Glass (Supplementary Fig. 1) accounts for one of the most promising smart devices concept, relying on a hands-free computing system with accurate voice control and imaging capabilities to enable on-the-fly human-machine interactions. The built-in wireless functions (Wi-Fi and Bluetooth) potentiate mobility by connecting directly (Wi-Fi) or indirectly (*via* Bluetooth-paired smartphone/tablet). These advantages have proven the Google Glass useful for a range of biological/biomedical applications including plant disease detection^42^, remote surgical communications and image-guided surgery^43^, diabetes management^44^, and point-of-care diagnostics^45^.

Here, we demonstrate for the first time a Google Glass-directed monitoring and control of microfluidic biosensors and actuators using a set of integrated sensors, hardware, software, and Glassware. Using a liver- and heart-on-a-chip systems as a model platform, we demonstrated seamless transmission of biosensor data onto the Google Glass for on-demand visualization and analysis of *i*) temperature and pH values, *ii*) microscopic observations (*e.g.* morphology of liver/cardiac organoids and video of beating cardiac organoids), and *iii*) on-Glass analysis (*e.g.* beating rate). Moreover, we showed our capability to selectively actuate microfluidic pneumatic valves and reservoirs to study the effects of pharmaceutical compounds on liver organoids in a primary human liver-on-a-chip platform. The Glass might be of particular importance in cases where the experimental conditions threaten human life, as when researchers work with highly contagious bacteria and virus or radioactivity. We believe that our Google Glass-based platform for monitoring and control will find widespread applications in biomedicine and may be further expanded to healthcare and environmental analysis where telemetry, remote control, and on-demand human-machine interactions are required in a safer manner.

## Results and Discussion

### Hardware, software, and Glassware for data transmission

Wearable devices allow us to interact in new ways with data and enable us to take fast decision-making actions. When designing such wearable technological solutions it is ideal that the interaction is a two-way process – one should be able to see new data and take appropriate action through the wearable that translates into tangible changes in the system. With this aim, we designed a Glassware solution to monitor and control microfluidic and organs-on-a-chip systems, aided by a set of custom-developed hardware and software.

**Figure 1:**
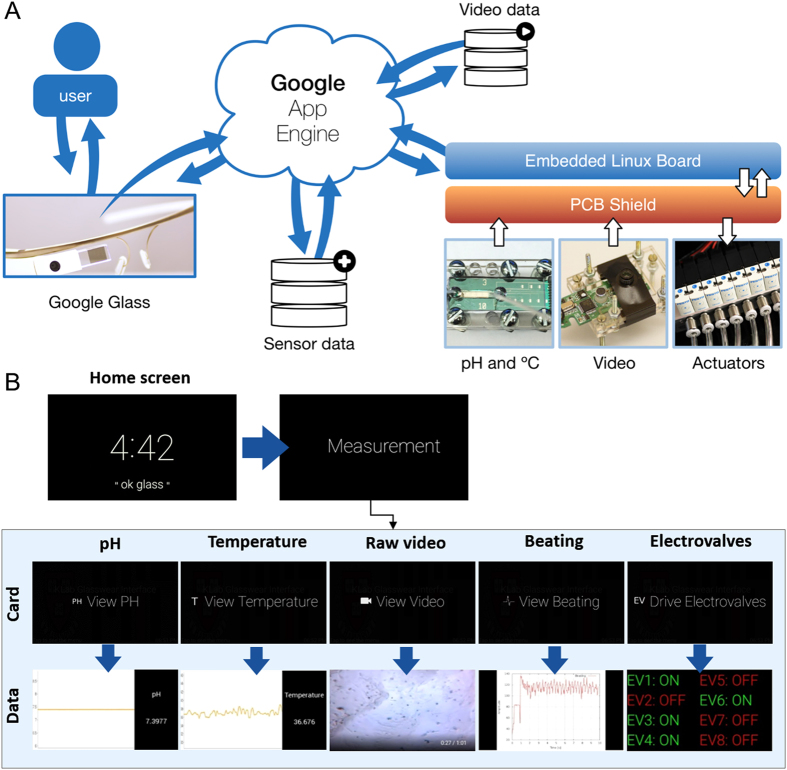
Principle of Google Glass-directed monitoring of sensor data and control of actuators. (**A**) Scheme of the interactions between the Google Glass and the hardware and software components. (**B**) Diagrams showing the operation procedure on the Google Glass. Upper panel shows the home screen, which upon voice control (“ok glass”) or tapping brings out the custom Card Measurement and enters the application; lower panel shows a series of Live Cards can then be reached by swiping. Tapping on each Card shows the corresponding measurement data, beating analysis, or the control card for the electrovalves.

The Google Glass communicates with the sensors *via* the Google App Engine (Fig. 1A). Data is stored in the Google Cloud, and the Glass periodically checks for new sensor data, such as the latest video of cardiomyocyte beating. A custom-built printed circuit board (PCB) shield (Supplementary Fig. 2) interfaces the sensors and the actuators (electrovalves) to an embedded Linux board (BeagleBone Black). This board was chosen for its low-cost, powerful processor, and high number (in total 92) of pins that allow the connection of multiple sensors and controllers. Furthermore, the board, programmed using C++, uses the Open Source Computer Vision (OpenCV) library to plot beating patterns from cardiomyocyte videos, based on our previously published work on the pixel-shift method^46^,^47^. To transmit data from the Beagle Bone to the Google App Engine we used the cURL library with simple *http post* and *http get* operations.

The voice control command (“ok glass”) gives the users access to the designed Glassware custom card (Measurement, Fig. 1B). Once the Glassware is launched, a set of Live Cards can be accessed *via* swiping, giving the user access to view the pH and temperature values, a video of the cells, and a plot of the beating patterns (Fig. 1B, bottom panel). Additionally, the user has access to a Live Card that actuates electrovalves, which can control the flow direction and addition of drugs in the microfluidic system. For the electrovalves a driver circuit was specifically designed to meet their voltage and current needs, and was controlled *via* digital output signals from the BeagleBone board. We have also designed custom circuits for temperature and pH sensors (Supplementary Fig. 2) in order to amplify the signals and reduce the noise of the sensors.

Overall, the sensor data is stored in the Google App Engine, and retrieved whenever the Glassware application is started and then refreshed periodically. Video files are also stored locally in a computer, so that data from any different time-point can be retrieved when needed. To actuate the different electrovalves, the Google Glass changes the status (on/off) of each one of the electrovalves in the Google App Engine, and the Linux board periodically checks for these status and makes concordant changes. Together, the developed platform allows the Glassware to update the user on data coming from the sensors and enable the user to take action through the control of electrovalves.

### Physical sensing units for real-time temperature and pH monitoring

**Figure 2:**
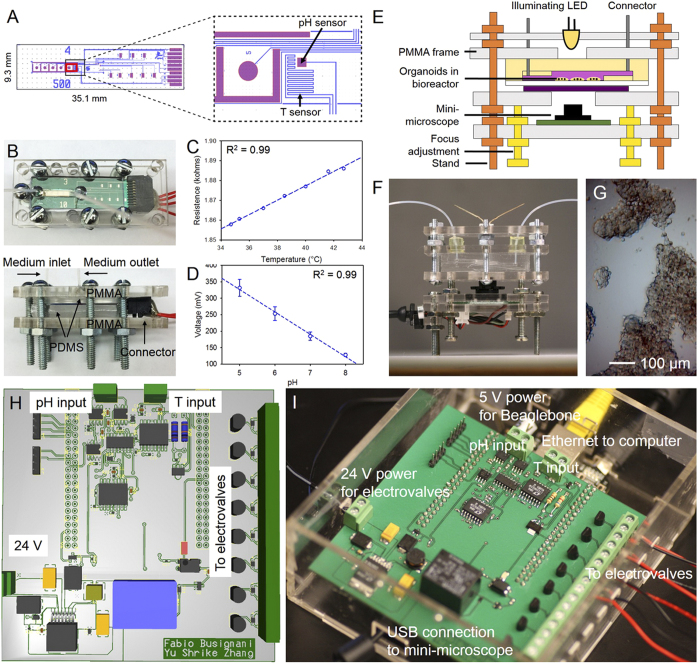
Biosensors and hardware for data recording, transmission, and command control. (**A**) Schematic showing the microfabricated biosensor chip containing temperature sensor, electrochemical pH sensor, and immunosensor array. (**B**) A microfluidic device hosting the biosensor chip for continuous sensing. (**C**,**D**) Calibration curves for temperature and pH sensors, respectively. (**E**,**F**) Schematic and photograph showing the mini-microscope. (**G**) Resolution testing of the mini-microscope. (**H**) Schematic showing the design of the electronic circuit for temperature/pH reading from the sensor and controlling electrovalves. (**I**) Photograph showing the assembled platform of the electronic circuit (top) and a BeagleBone board (bottom) for simultaneous reading of the sensor data, communication with the mini-microscope, and control over electrovalves.

Microfabricated sensors were obtained using our published miniaturized approach^48^. Figure 2A shows a schematic of the sensor array measuring 2.2 × 15 mm^2^, which hosts five biosensor platforms, a temperature sensor, and a pH electrode. The entire sensor array was fabricated from biocompatible materials and integrated with a complementary metal-oxide semiconductor (CMOS) chip for measurements. As a proof of principle, we chose to only probe the temperature and pH responses of the sensor array to characterize the physical microenvironment of our system. The right panel in Fig. 2A indicates a magnified view of the temperature and pH sensing units of the sensor array, where the temperature sensor consisted of a platinum (Pt) zigzagging path of 0.02 × 16 mm^2^ for each turn. Pt was chosen due to its linearity within the physiological temperature range and higher resistivity, which efficiently confined the size of the sensor to a small footprint^49^. The pH sensor consisted of a 250-μm^2^ metal pad that was electrodeposited with a thin film of iridium oxide (IrOx). Changes in the pH of the surrounding medium were measured by open circuit potential of the electrode^50^,^51^. To achieve continual monitoring of the microenvironment, the sensor array was enclosed in a microfluidic device, with a 3 × 15 × 0.2 mm^3^ chamber placed directly on top of the sensing units, resulting in a small working volume of <10 μL (Fig. 2B). Calibration curves for temperature and pH utilizing the enclosed microfluidic device show a linear correlation, and a sensitivity of 3.6 Ω °C^−1^ and −67.9 mV pH^−1^ at the flow rate of 200 μm h^−1^ (Fig. 2C,D).

We further integrated a miniature microscope that we have recently developed for image and video acquisition^46^,^47^,^52^. The microscope was fabricated from a webcam and off-the-shelf components^46^. Figure 2E,F show a schematic and a photograph of the mini-microscope integrated with a microfluidic bioreactor, respectively. The imaging unit was constructed by flipping the webcam lens and re-attaching it to the CMOS sensor in order to achieve magnification rather than the de-magnifying mechanism that a webcam requires^46^. This imaging unit was fitted on a set of poly(methyl methacrylate) (PMMA) frames sandwiched by screws/bolts and the microfluidic bioreactor placed above the sensor. A mini-microscope image of HepG2 cells in a liver bioreactor is shown in Fig. 2G, where individual cells could be observed, highlighting the high resolution of the mini-microscope.

In order to transmit the data to the Google App Engine a custom PCB board was designed to accommodate the BealgeBone Black for connection with the pH and temperature sensors as well as the mini-microscope (Fig. 2H,I). The micro-computing unit then records the data and image/videos and constantly transmits them remotely to the Cloud for the Glass to fetch and display.

### Remote monitoring of liver- and heart-on-a-chip platforms

**Figure 3:**
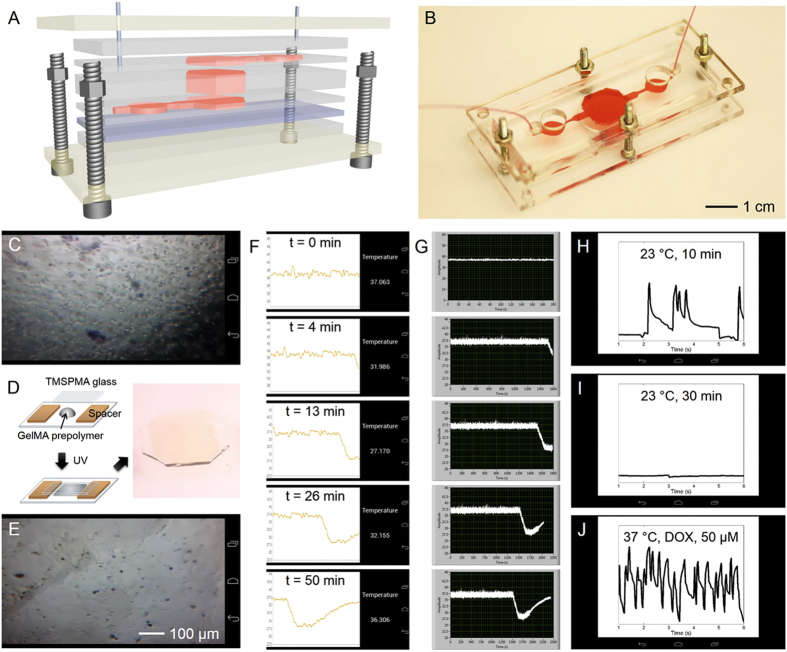
Real-time monitoring of organoid behaviors in an integrated liver-and-heart-on-a-chip platform on Google Glass. (**A**,**B**) Schematic and photograph showing the resealable microfluidic bioreactor. The mini-microscopes were fitted at the bottom of the bioreactors while the biosensor unit was placed downstream of the bioreactors. (**C**) Google Glass view obtained from the mini-microscope fitted underneath the liver bioreactor showing the morphology of HepG2 cells. (**D**) Schematics showing the fabrication process of a GelMA substrate for constructing the cardiac tissue. The cardiac tissue was then transferred into the cardiac bioreactor. (**E**) Google Glass view obtained from the mini-microscope fitted underneath the cardiac bioreactor showing the morphology of rat cardiomyocytes. (**F**) Temperature sensing data visualized on the Google Glass, where the door of the incubator was opened for 10 min and then closed. (**G**) The same data was recorded on a LabVIEW program, indicating the same trend and accuracy of the data transmitted onto the Google Glass. (**H–J**) Beating analysis on the Google Glass of the cardiomyocytes under different conditions: (**H**) 23 °C for 10 min; (**I**) 23 °C for 30 min; and (**J**) 37 °C post treatment of DOX for 1 h.

To test the capability of our Google Glass system for visualizing sensor data we monitored liver- and heart-on-a-chip platforms. The multi-layer microfluidic bioreactor was fabricated following our recently published protocol (Fig. 3A,B)^10^, where the bioreactor chambers were made of polydimethylsiloxane (PDMS). The bioreactor was designed to be re-usable and re-sealable, providing easy access to seeding cells or placing organoids inside the chamber. To construct the liver bioreactor we seeded HepG2 cells in the bottom chamber at a density of approximately 1000 cells mm^−2^. The mini-microscope was fitted at the bottom of the culture chamber for continuous monitoring of cell behavior. The images acquired from the mini-microscope were successfully transmitted to the Google Glass wirelessly and visualized in the View Image Live Card (Fig. 3C). The mini-microscope images can be acquired at a pre-set time interval and saved locally on the computer. Importantly, the latest acquired image is wirelessly transmitted to the Google Glass, where it then refreshes the Live Card for visualization. This feature enables the user to remotely access the microscopic images of the organoids in the bioreactors and monitor their morphological changes as a function of time.

However, in many cases, static images cannot be used to follow and measure fast dynamic cellular behaviors. We next demonstrated the capability of the Google Glass to simultaneously visualize and analyze mini-microscope videos using a simplified heart-on-a-chip platform. Heart-on-a-chip provides a versatile approach for studying the biology, physiology, as well as screening pharmaceutical compounds possessing cardiotoxicity^26^,^53^,^54^. The heart-on-a-chip platform was constructed by seeding rat neonatal cardiomyocytes on a piece of glass coated with a thin layer of 5 wt.% gelatin methacryloyl (GelMA) and 1 mg mL^−1^ carbon nanotubes (CNTs), followed by placement of the construct in the chamber of a cardiac bioreactor (Fig. 3D). CNTs were embedded into the GelMA to promote the intercellular connections among the cardiomyocytes, therefore improving the functionality of the fabricated cardiac organoids^55^,^56^. Figure 3E is the View Image Live Card showing a mini-microscope image of the cardiomyocytes in the cardiac bioreactor transmitted to the Google Glass, indicating the formation of a confluent layer of cardiomyocytes on the GelMA/CNTs substrate.

Neonatal cardiomyocytes beat synchronously, but the beating frequency and pattern can be disturbed easily by administration of drugs/toxins or by changing the surrounding microenvironment (*e.g.* temperature). We therefore performed two sets of experiments to perturb the regular beating of the cardiac organoid in the heart-on-a-chip device. We first opened the incubator door for 10 min to allow the temperature to drop from 37 °C to 27 °C, and then closed it back. The temperature sensor detected the drop in real-time (Fig. 3F), and the data was transmitted *via* the integrated system to the Google Glass and visualized in the View Temperature Live Card. The temperature data was consistent with that collected by a commercial sensor directly connected with a National Instruments data acquisition (NI-DAQ) card and LabVIEW (Fig. 3G), highlighting the accuracy and time responsiveness of the sensor data transmitted to the Google Glass. The beating of the cardiac organoid was monitored during the external manipulation of the temperature, recorded using the mini-microscope and the video wirelessly transmitted to the Google Glass (Supplementary Movie 1). When the heart-on-a-chip temperature decreased to 23 °C, even for only 10 min, the cardiomyocytes showed an irregular and reduced beating rate, which was analyzed and plotted on the Google Glass in the View Beating Live Card (Fig. 3H). Furthermore, when the cardiac bioreactor was completely removed from the incubator and cooled to room temperature for over 30 min, the cardiomyocytes completely ceased beating (Supplementary Movies 2 and Fig. 3I). Alternative to changing environmental conditions, the beating of the cardiac organoid was also tested by addition of cardiotoxic drugs. We infused the heart-on-a-chip with 50 μM of doxorubicin (DOX) for 1 h at 37 °C and monitored the beating. DOX, an anti-cancer drug with adverse side effects on cardiac tissues, was shown to induce acute arrhythmia of the cardiac organoid upon treatment at high doses (Supplementary Movie 3 and Fig. 3J)^26^,^47^,^57^,^58^.

The integrated BeagleBone board and software/Glassware sets allowed the data from the microfabricated sensor, as well as still images and videos recorded by the mini-microscope, to be visualized and transmitted wirelessly to the Google Glass. The sensing capabilities of our system was enhanced with real-time analysis and concomitant visualization in the Google Glass. Importantly, the data were simultaneously recorded locally (on the computer where the BeagleBone board was connected), facilitating on-demand retrieval of the data at any time. In addition to organoids in bioreactors, a range of other static images or dynamic videos such as micropatterns and microfluidic droplet generation could also be remotely monitored in real-time using the Google Glass (Supplementary Fig. 4 and Movie 4).

### Remote control of actuators using Google Glass

**Figure 4:**
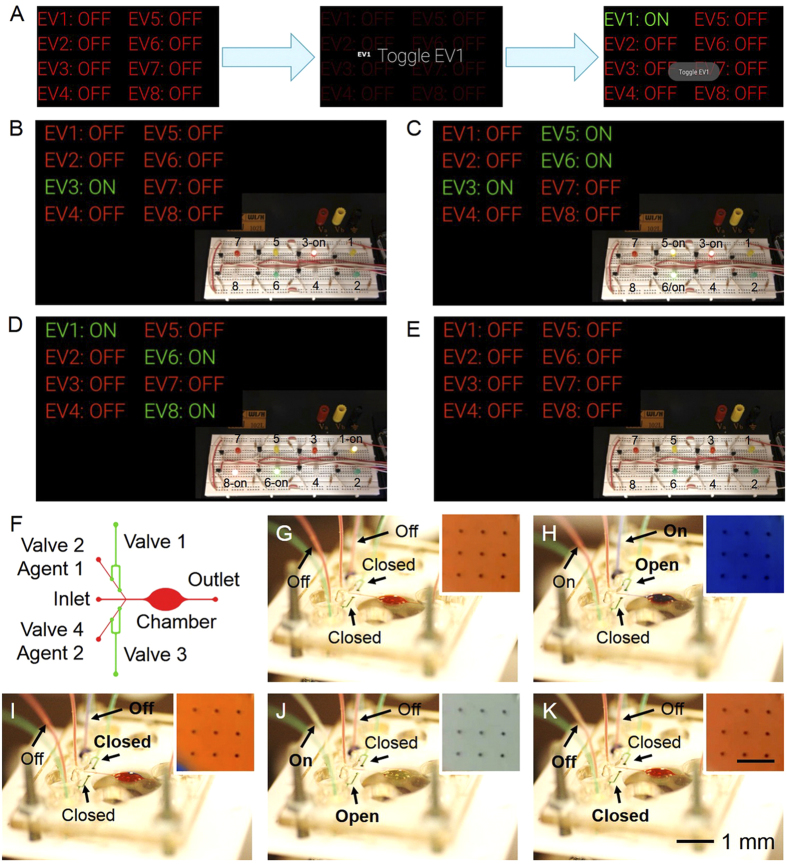
Controlling electrovalves and microfluidic actuators using Google Glass. (**A**) Diagrams showing the control of the electrovalves on the Google Glass. (**B–E**) Demonstration of control over the blinking of LEDs from the Glass. (**E**) A microfluidic bioreactor with built-in valves and inlets for drug testing. A mini-microscope was fitted at the bottom of the bioreactor for real-time analysis. (**F**) Schematic of the microfluidic bioreactor for evaluating the Google Glass-directed electrovalve controllers. (**F–K**) Sequential activation of Valve 1 and Valve 2 from the Glass, as indicated by alternation of dyes injected from the inlet, Agent 1 channel, and Agent 2 channel. Insets show images taken by the mini-microscope at the bottom of the bioreactor chamber clearly indicating matching color changes in the flow.

Actuators play a pivotal role in microdevices, functioning as gating mechanisms for controlling a variety of devices based on electricity and mechanics^59^,^60^. For example, electrovalves are a category of electrically actuated valves that allow for opening and closure of pressure-driven valves that can be used to conveniently manipulate liquid flows inside a microfluidic device. Here we have developed a remote control platform where the BeagleBone board reads the wirelessly transmitted Google Glass commands and responds to actuate the electrovalves (Fig. 1A). The Glassware application has a set of Live Cards that allow turning on and off each electrovalve switch upon command (Fig. 4A). Selection of the “Drive Electrovalves” Card enables a list of eight valves in the screen of the Glass; upon swiping of the touch pad eight Live Cards will be sequentially shown, each of which can be individually triggered. To visually show the working concept, we prepared an array of eight LEDs connected to the outputs on the BeagleBone board (Fig. 2H,I). As shown in Fig. 4B–E, when the switches were selectively activated on the Google Glass the corresponding LEDs could be turned on and off. The capabilities to sequentially activate and deactivate LEDs and random manipulation are further shown in Supplementary Movies 5 and 6.

We subsequently constructed a microfluidic bioreactor consisting of an elliptical chamber, one central inlet for medium circulation, and two side inlets with corresponding pressure-driven pneumatic valves, which together with linked pressure-driven reservoirs (both activated by electrovalves), accomplish the injection of target agents (Fig. 4F). We then first demonstrated the capability to remotely actuate the valves with the commands wirelessly transmitted from the Google Glass to the BeagleBone board. As shown in Fig. 4G–K and Supplementary Movie 7, we initially injected medium in the central inlet, with both side channels closed by the valves (Fig. 4G). To drive blue dye from the reservoir to the bioreactor chamber we sequentially opened the channel by deactivating Valve 1 and pressurized the blue dye reservoir by activating Valve 2 (Fig. 4H). Reversing these actions and performing the same Google Glass commands on Valves 3 and 4 drove yellow dye to infuse the bioreactor chamber (Fig. 4J). Finally, the valves were reset to the initial configuration, restoring the circulation of the medium through the central inlet (Fig. 4K).

Additionally, a mini-microscope was fitted at the bottom of the bioreactor for real-time monitoring, and connected to the BeagleBone board. The insets in Fig. 4F–J, show the change in color of the liquid pumped into the bioreactor chamber, monitored by the mini-microscope in real-time. The dynamic process of synchronized mini-microscope recording, the activation of the valves, and injection of reagents into the bioreactor, together with the commands on the Google Glass can be observed in Supplementary Movie 7.

### Simultaneous remote control and monitoring of liver-on-a-chip for drug testing

**Figure 5:**
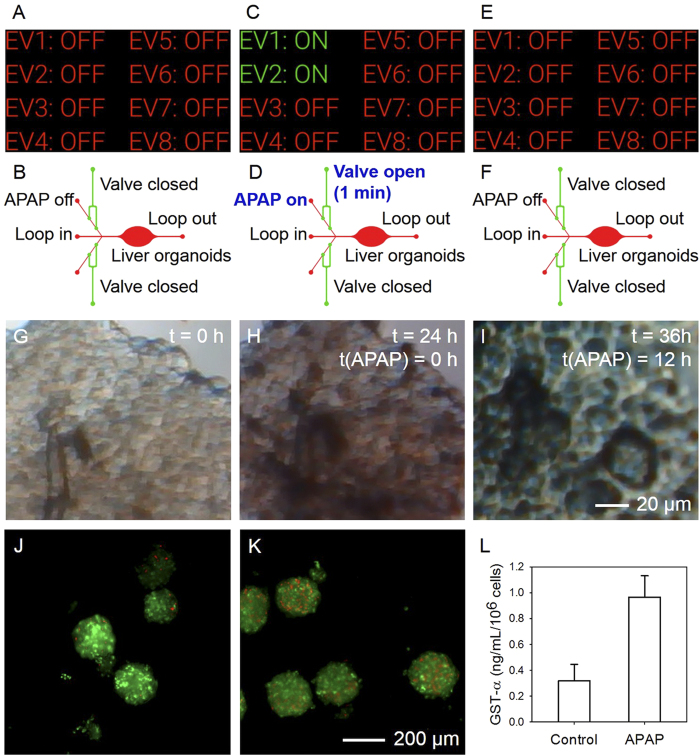
Remote activation of electrovalves using the Google Glass for drug testing on the liver-on-a-chip platform fabricated from human primary hepatocyte spheroids. (**A**,**B**) All valves were off to allow regular culture of the liver organoids. (**C**,**D**) At 24 h post culture, Valves 1/2 were activated from the Glass and 15 mM APAP was injected into the circulation for 1 min. (**E**,**F**) Valves 1/2 were then deactivated and the culture was maintained for another 12 h. (**G**–**I**) Mini-microscopic image clearly showed swelling, sign of apoptosis of the liver organoid post APAP treatment. (**J**,**K**) Live/dead assay of the liver organoids (**J**) with and (**K**) without APAP treatment, indicating increased cell death when the cells were incubated with APAP. (**L**) Levels of the liver damage biomarker GST-α measured by ELISA, showing the same trend of cell death when treated with APAP.

The main purpose of the liver is to provide detoxification of various metabolites, protein/enzyme synthesis, and the production of bile necessary for digestion of food, rendering hepatotoxicity studies an important target for multiple fields. Here we introduced human primary hepatocytes into the chamber of the microfluidic bioreactor to construct a liver-on-a-chip platform. Liver spheroids of approximately 200 μm were first formed using a non-adherent microwell array^61^, which were then retrieved, suspended in GelMA, and crosslinked to the bottom of the bioreactor chamber. Prior to the experiment, 15 mM acetaminophen (APAP), a hepatotoxic drug, was added to the reservoir of one of the side channels. The liver bioreactor was initially perfused with hepatocyte culture medium from the central inlet in the first 24 h with the side channels closed (Fig. 5A,B). At 24 h we used the Google Glass to inject APAP for 1 min. Commands were sent from the Glass deactivating the valve and activating the pressure to inject APAP (Fig. 5C,D). The channel was then closed to stop the APAP injection and restore the regular perfusion with the hepatocyte growth medium (Fig. 5E,F). The mini-microscope fitted at the bottom of the bioreactor monitored the morphology of the liver spheroids, transmitting the data to the Google Glass for real-time, *in situ* monitoring of drug treatment effect. Without any drug, the liver organoid remained healthy and tightly agglomerated (Fig. 5G,H). In comparison, 12 h post injection of 15 mM APAP, the liver spheroid micrographs transmitted through the mini-microscope to the Google Glass showed swollen cellular structures (Fig. 5I), clearly indicating signs of a toxic response to APAP treatment. The decreased liver functionality was further confirmed by off-chip viability assay and analysis of damage biomarker glutathione S-transferase *α* (GST-*α*, Fig. 5J–L), well correlating to the observed hepatotoxicity with the Google Glass-directed drug administration and organoid monitoring.

In this particular demonstration we highlight that the entire process, including the operation of the valves, injection of the drug, restoration of the main perfusion, and monitoring the morphology with the mini-microscope was solely controlled by the Google Glass without direct manipulation of the liver-on-a-chip device or the valves system. Such seamless interface allows for remote actuation of microfluidic devices and easy access of sensed data, potentially enabling long-term communication and control between humans and microdevices.

## Conclusions

For the first time we have developed a set of hardware, firmware, and Glassware that enabled wireless transmission of sensor data onto the Google Glass for on-demand data visualization and real-time analysis. We have further engineered the hardware and software to allow the control of electrical outputs from commands entered by the Glass. We demonstrated the capability of the platform to monitor physical and physiological parameters such as temperature, pH, and microscopic morphological images and videos, of an integrated liver-and-heart-on-a-chip. The Glass also achieved beating analysis of the cardiac organoids. We then proved that the pneumatic electrovalves could be remotely activated by the Glass to introduce pharmaceutical compounds into the microfluidic liver bioreactor at desired time points, with their effects being transmitted to the Glass for continuous real-time monitoring.

We believe that such an innovative platform is a premise in wearable sensing and controlling technology for applications in biomedicine, surgery and general laboratory. The Glass might be of particular importance in cases where the experimental conditions threaten human life, as when researchers work with highly contagious bacteria and virus or radioactivity.

## Methods

### Microfabricated temperature and temperature sensors

The passive chip was microfabricated with a two-mask process flow. Silicon wafers with a layer of native oxide 500 nm in thickness were used as the substrate. The metallization of the wafers was achieved by first evaporation of 10 nm of titanium (Ti) as the adhesive layer, followed by 100 nm of Pt on top of Ti. Passivation of the metal was performed *via* atomic layer deposition of Al_2_O_3_ of 20 nm in thickness^62^. The fabrication process is shown Supplementary Fig. 2. The pH sensor was functionalized by electrodeposition of IrOx onto the microelectrode, which was carried out by applying a constant current density of 0.15 mA cm^-2^ for 80 min in a solution of IrOx^63^. The pH was measured by reading the open circuit potential produced by the electrodes using a potentiostat (CHI684, CH instruments) and an external Ag/AgCl reference electrode (ET072-1, eEAQ). Temperature sensing was achieved by reading the sensor resistance using a precision source meter (B2901A, Agilent).

### Software and Glassware

The Google Glass Explorer Edition was programmed using the Glass Development Kit in Java. The embedded Linux board BeagleBone Black was programmed in C++ and bash command. OpenCV was used to calculate the beating rate of cardiomyocytes and cURL library was used to perform *http* *post* and *get* operations to the Google App Engine. The cloud-based Google App Engine, programmed in Python, and local video storing was done using the Qt framework.

### Design of printed circuit board (PCB)

A custom made PCB was designed using the National Instruments Circuit Design Suite, to accommodate the pH/temperature multisensor and the electrovalves driver and modified to attach the headers of the BeagleBone Black (Fig. S3). A conditioning circuit for both pH and temperature sensors was designed to provide noise filtering with a low-pass filter. Additionally, the pH circuit performed a signal level shift, due to the fact that the pH sensor signals are bipolar. To drive the electrovalves (normally open, MH1-A-24VDC-N-HC-8V-PR-K01-QM-AP-BP-CX-DX, Festo), a specific circuit was designed with a low-side switch metal-oxide semiconductor (MOS). A DC-DC converter circuit was designed to provide compatibility with both 24 V and 12 V solenoid valves, and a relay circuit (voltage supply switch) provided the switch between these. Circuit designs followed the guidelines for reduced electromagnetic interference.

### Mini-microscope

A commercial Logitech C160 webcam was dissembled to retrieve the complementary metal-oxide semiconductor (CMOS) sensor. We then inverted the lens to achieve magnification and convert the webcam into the mini-microscope^52^. The base structure of the mini-microscope was fabricated from a PMMA sheet by laser cutting (VLS 2.30 Desktop Laser System, Universal Laser Systems), following our recently published protocol^46^.

### Liver- and heart-on-chips

Re-usable and re-sealable microfluidic bioreactors based on polydimethylsiloxane (PDMS, Dow Corning Sylgard 184, Ellsworth) were fabricated by modifying our recently developed protocol^10^. Specifically, the bioreactors had a double-layer configuration with the inlet and organoid chamber at the bottom and outlet on the top layer. The two PDMS layers could be disassembled for seeding the organoid, following which sealing were achieved by clamping the pieces using a pair of PMMA structures tightened by sets of screws/bolts.

HepG2/C3A human hepatocellular carcinoma cells (HB-8065, ATCC) were seeded onto the bottom of the bioreactors at a density of 2000 cells mm^−2^. Cultures of the organoids were maintained at a constant flow rate of 200 μL h^−1^ in a Dulbecco’s Modified Eagle’s Medium (DMEM, Life Technologies) supplemented with 10 vol.% fetal bovine serum (FBS, Life Technologies) and 1 vol.% penicillin-streptomycin (P/S, Life Technologies). Primary human hepatocytes (HUCPI6, Triangle Research Labs, Research Triangle Park, NC) were seeded in a PDMS model containing arrays of 200-μm wells. To maintain the high cell viability, the hepatocyte growth media (Lonza) was replaced every 48 h. At Day 5 the spheroids were harvested and mixed with an aqueous solution containing 10 w/v% GelMA and 0.5 w/v% photo initiator Irgacure 2959 (Ciba, Hawthorne, NY). GelMA droplets with approximately 1 mm in size were bioprinted in the bioreactor chamber using a NovoGen MMX bioprinter (Organovo, San Diego, CA). After 17 s of UV exposure at 850 mW at a distance of 8.5 cm, the GelMA dots were successfully crosslinked to the bottom of the bioreactor^10^. When necessary, APAP was introduced into the bioreactor at a concentration of 15 mM.

Cardiac bioreactors were constructed from neonatal rat cardiomyocytes, which were isolated from Sprague-Dawley rats of 2 days old following our established protocol approved by the Institutional Animal Care and Use Committee^64^. The cardiomyocytes were then seeded onto GelMA hydrogel mats (6 × 6 mm^2^) containing 1 mg mL^−1^ CNTs at a number of 5 × 10^5^ cells^56^. The GelMA-CNT mats seeded with cardiomyocytes were initially cultured in DMEM supplemented with 10 vol.% FBS, 1 vol.% P/S, and 1 vol.% L-glutamine (Life Technologies) for 3 days until consistent beating of the cells was observed. The constructs were subsequently transferred to the chambers of the bioreactors and perfusion-cultured at a flow rate of 200 μL h^−1^.

## Additional Information

**How to cite this article**: Zhang, Y. S. *et al.* Google Glass-Directed Monitoring and Control of Microfluidic Biosensors and Actuators. *Sci. Rep.* **6**, 22237; doi: 10.1038/srep22237 (2016).

## Supplementary Material

Supplementary Information

Supplementary Movie 1

Supplementary Movie 2

Supplementary Movie 3

Supplementary Movie 4

Supplementary Movie 5

Supplementary Movie 6

Supplementary Movie 7

## References

[b1] Nardini C. *et al.* i-Needle: Detecting the biological mechanisms of acupuncture. Science 346, S21–S22 (2014).

[b2] Ottesen E. A., Hong J. W., Quake S. R. & Leadbetter J. R. Microfluidic digital PCR enables multigene analysis of individual environmental bacteria. Science 314, 1464–1467 (2006). 17138901 10.1126/science.1131370

[b3] Groisman A., Enzelberger M. & Quake S. R. Microfluidic memory and control devices. Science 300, 955–958 (2003). 12738857 10.1126/science.1083694

[b4] Thorsen T., Maerkl S. J. & Quake S. R. Microfluidic large-scale integration. Science 298, 580–584 (2002). 12351675 10.1126/science.1076996

[b5] Unger M. A., Chou H.-P., Thorsen T., Scherer A. & Quake S. R. Monolithic microfabricated valves and pumps by multilayer soft lithography. Science 288, 113–116 (2000). 10753110 10.1126/science.288.5463.113

[b6] Beebe D. J., Mensing G. A. & Walker G. M. Physics and applications of microfluidics in biology. Annu Rev Biomed Eng 4, 261–286 (2002). 12117759 10.1146/annurev.bioeng.4.112601.125916

[b7] Stone H. A., Stroock A. D. & Ajdari A. Engineering flows in small devices: microfluidics toward a lab-on-a-chip. Annu Rev Fluid Mech 36, 381–411 (2004).

[b8] Whitesides G. M. The origins and the future of microfluidics. Nature 442, 368–373 (2006). 16871203 10.1038/nature05058

[b9] Zhang Y. S. & Khademhosseini A. Seeking the Right Context for Evaluating Nanomedicine: from Tissue Models in Petri Dishes to Microfluidic Organs-on-a-Chip. Nanomedicine 10, 685–688 (2015). 25816872 10.2217/nnm.15.18

[b10] Bhise N. S. *et al.* A Liver-on-a-Chip Platform with Bioprinted Hepatic Spheroids. Biofabrication 8, 014101 (2016). 26756674 10.1088/1758-5090/8/1/014101

[b11] Wikswo J. P. The relevance and potential roles of microphysiological systems in biology and medicine. Experimental Biology and Medicine 239, 1061–1072 (2014). 25187571 10.1177/1535370214542068 PMC4330974

[b12] Sei Y., Justus K., LeDuc P. & Kim Y. Engineering living systems on chips: from cells to human on chips. Microfluid Nanofluid, 1–14 (2014).

[b13] Polini A. *et al.* Organs-on-a-chip: a new tool for drug discovery. Expert Opin Drug Discov 9, 335–352 (2014). 24620821 10.1517/17460441.2014.886562

[b14] Moya M. L. & George S. C. Integrating *in vitro* organ-specific function with the microcirculation. Curr Opin Chem Eng 3, 102–111 (2014). 10.1016/j.coche.2013.12.004 PMC3979533 24729953

[b15] Ebrahimkhani M. R., Young C. L., Lauffenburger D. A., Griffith L. G. & Borenstein J. T. Approaches to *in vitro* tissue regeneration with application for human disease modeling and drug development. Drug Discov Today 19, 754–762 (2014). 24793141 10.1016/j.drudis.2014.04.017 PMC4104173

[b16] Bhise N. S. *et al.* Organ-on-a-chip platforms for studying drug delivery systems. J Controlled Release 190, 82–93 (2014). 10.1016/j.jconrel.2014.05.004 PMC4142092 24818770

[b17] Bhatia S. N. & Ingber D. E. Microfluidic organs-on-chips. Nat Biotechnol 32, 760–772 (2014). 25093883 10.1038/nbt.2989

[b18] Wikswo J. P. *et al.* Scaling and systems biology for integrating multiple organs-on-a-chip. Lab Chip 13, 3496–3511 (2013). 23828456 10.1039/c3lc50243k PMC3818688

[b19] Selimović Š., Dokmeci M. R. & Khademhosseini A. Organs-on-a-chip for drug discovery. Curr Opin Pharmacol 13, 829–833 (2013). 23850526 10.1016/j.coph.2013.06.005

[b20] Moraes C., Mehta G., Lesher-Perez S. C. & Takayama S. Organs-on-a-chip: a focus on compartmentalized microdevices. Ann Biomed Eng 40, 1211–1227 (2012). 22065201 10.1007/s10439-011-0455-6

[b21] Ghaemmaghami A. M., Hancock M. J., Harrington H., Kaji H. & Khademhosseini A. Biomimetic tissues on a chip for drug discovery. Drug Discov Today 17, 173–181 (2012). 22094245 10.1016/j.drudis.2011.10.029 PMC3273552

[b22] Huh D., Hamilton G. A. & Ingber D. E. From 3D cell culture to organs-on-chips. Trends Cell Biol 21, 745–754 (2011). 22033488 10.1016/j.tcb.2011.09.005 PMC4386065

[b23] Ebrahimkhani M. R., Neiman J. A. S., Raredon M. S. B., Hughes D. J. & Griffith L. G. Bioreactor technologies to support liver function *in vitro*. Adv Drug Del Rev 69, 132–157 (2014). 10.1016/j.addr.2014.02.011 PMC4144187 24607703

[b24] Wikswo J. P. *et al.* Engineering Challenges for Instrumenting and Controlling Integrated Organ-on-Chip Systems. IEEE Transact Biomed Eng 60, 682–690 (2013). 10.1109/TBME.2013.2244891 PMC3696887 23380852

[b25] Eklund S. E. *et al.* Metabolic discrimination of select list agents by monitoring cellular responses in a multianalyte microphysiometer. Sensors 9, 2117–2133 (2009). 22574003 10.3390/s90302117 PMC3345856

[b26] Zhang Y. S. *et al.* From Cardiac Tissue Engineering to Heart-on-a-Chip: Beating Challenges. Biomed Mater 10, 034006 (2015). 26065674 10.1088/1748-6041/10/3/034006 PMC4489846

[b27] Stradolini S. *et al.* Wireless Monitoring of Endogenous and Exogenous Biomolecules on an Android Interface. IEEE Sens J (2015).

[b28] Laksanasopin T. *et al.* A smartphone dongle for diagnosis of infectious diseases at the point of care. Sci Transl Med 7, 273re271–273re271 (2015). 10.1126/scitranslmed.aaa0056 25653222

[b29] Im H. *et al.* Digital diffraction analysis enables low-cost molecular diagnostics on a smartphone. Proct Natl Acad Sci USA 112, 5613–5618 (2015). 10.1073/pnas.1501815112 PMC4426451 25870273

[b30] Vashist S. K., Mudanyali O., Schneider E. M., Zengerle R. & Ozcan A. Cellphone-based devices for bioanalytical sciences. Anal Bioanal Chem 406, 3263–3277 (2014). 24287630 10.1007/s00216-013-7473-1 PMC4024356

[b31] Petryayeva E. & Algar W. R. Multiplexed homogeneous assays of proteolytic activity using a smartphone and quantum dots. Anal Chem 86, 3195–3202 (2014). 24571675 10.1021/ac500131r

[b32] Liang P.-S., San Park T. & Yoon J.-Y. Rapid and reagentless detection of microbial contamination within meat utilizing a smartphone-based biosensor. Sci Rep 4 (2014). 10.1038/srep05953 PMC4121612 25092261

[b33] Giller G. Using a Smartphone to Detect Cancer. Sci Am 310, 28–28 (2014).

[b34] Olivo J. *et al.* Android Interface for Wireless Monitoring of Cell Cultures. Proc Int IEEE Conf BioCAS pp 400–403 (2014).

[b35] Gallegos D. *et al.* Label-free biodetection using a smartphone. Lab Chip 13, 2124–2132 (2013). 23609514 10.1039/c3lc40991k

[b36] Khatua S. & Orrit M. Toward single-molecule microscopy on a smart phone. ACS Nano 7, 8340–8343 (2013). 24112048 10.1021/nn405167q

[b37] Wei Q. *et al.* Fluorescent imaging of single nanoparticles and viruses on a smart phone. ACS Nano 7, 9147–9155 (2013). 24016065 10.1021/nn4037706 PMC3951925

[b38] Lam S. C. K., Wong K. L., Wong K. O., Wong W. & Mow W. H. A smartphone-centric platform for personal health monitoring using wireless wearable biosensors. IEEE Int Conf Info Commun Signal Processing, pp 1–7 (2009).

[b39] Windmiller J. R. & Wang J. Wearable electrochemical sensors and biosensors: a review. Electroanalysis 25, 29–46 (2013).

[b40] Shim B. S., Chen W., Doty C., Xu C. & Kotov N. A. Smart electronic yarns and wearable fabrics for human biomonitoring made by carbon nanotube coating with polyelectrolytes. Nano Lett 8, 4151–4157 (2008). 19367926 10.1021/nl801495p

[b41] Iguchi S. *et al.* A flexible and wearable biosensor for tear glucose measurement. Biomed Microdevices 9, 603–609 (2007). 17520370 10.1007/s10544-007-9073-3

[b42] Cortazar B., Koydemir H. C., Tseng D., Feng S. & Ozcan A. Quantification of plant chlorophyll content using Google Glass. Lab Chip 15, 1708–1716 (2015). 25669673 10.1039/c4lc01279h PMC4366296

[b43] Shao P. *et al.* Designing a Wearable Navigation System for Image-Guided Cancer Resection Surgery. Ann Biomed Eng 42, 2228–2237 (2014). 24980159 10.1007/s10439-014-1062-0 PMC4332818

[b44] Wall D., Ray W., Pathak R. D. & Lin S. M. A Google Glass Application to Support Shoppers With Dietary Management of Diabetes. J Diabetes Sci Technol 8, 1245–1246 (2014). 25015954 10.1177/1932296814543288 PMC4455467

[b45] Feng S. *et al.* Immunochromatographic diagnostic test analysis using Google Glass. ACS Nano 8, 3069–3079 (2014). 24571349 10.1021/nn500614k PMC3988681

[b46] Zhang Y. S. *et al.* A Cost-Effective Fluorescence Mini-Microscope with Adjustable Magnifications for Biomedical Applications. Lab Chip 15, 3661–3669 (2015). 26282117 10.1039/c5lc00666j PMC4550514

[b47] Kim S. B. *et al.* A cell-based biosensor for real-time detection of cardiotoxicity using lensfree imaging. Lab Chip 11, 1801–1807 (2011). 21483937 10.1039/c1lc20098d PMC3611966

[b48] Cavallini A. *et al.* A Subcutaneous Biochip for Remote Monitoring of Human Metabolism: packaging and biocompatibility assessment. IEEE Sensors J 15, 417–424 (2015).

[b49] Baker B. Temperature sensing technologies. AN679, Microchip Technology Inc (1998).

[b50] Weiland J. D., Anderson D. J. & Humayun M. S. *In vitro* electrical properties for iridium oxide versus titanium nitride stimulating electrodes. IEEE Transact Biomed Eng 49, 1574–1579 (2002). 10.1109/TBME.2002.805487 12549739

[b51] Olthuis W., Robben M., Bergveld P., Bos M. & Van der Linden W. pH sensor properties of electrochemically grown iridium oxide. Sensors Actuators B: Chem 2, 247–256 (1990).

[b52] Kim S. B. *et al.* A mini-microscope for *in situ* monitoring of cells. Lab Chip 12, 3976–3982 (2012). 22911426 10.1039/c2lc40345e PMC3601772

[b53] Agarwal A., Goss J. A., Cho A., McCain M. L. & Parker K. K. Microfluidic heart on a chip for higher throughput pharmacological studies. Lab Chip 13, 3599–3608 (2013). 23807141 10.1039/c3lc50350j PMC3786400

[b54] Grosberg A., Alford P. W., McCain M. L. & Parker K. K. Ensembles of engineered cardiac tissues for physiological and pharmacological study: heart on a chip. Lab Chip 11, 4165–4173 (2011). 22072288 10.1039/c1lc20557a PMC4038963

[b55] Shin S. R. *et al.* Carbon-Nanotube-Embedded Hydrogel Sheets for Engineering Cardiac Constructs and Bioactuators. ACS Nano 7, 2369–2380 (2013). 23363247 10.1021/nn305559j PMC3609875

[b56] Shin S. R. *et al.* Carbon nanotube reinforced hybrid microgels as scaffold materials for cell encapsulation. ACS Nano 6, 362–372 (2011). 22117858 10.1021/nn203711s PMC3401631

[b57] Zhang S. *et al.* Identification of the molecular basis of doxorubicin-induced cardiotoxicity. Nat Med 18, 1639–1642 (2012). 23104132 10.1038/nm.2919

[b58] Menna P., Salvatorelli E. & Minotti G. Cardiotoxicity of Antitumor Drugs†. Chem Res Toxicol 21, 978–989 (2008). 18376852 10.1021/tx800002r

[b59] Weibel D. B., Kruithof M., Potenta S., Sia S. K., Lee A. & Whitesides G. M. Torque-actuated valves for microfluidics. Anal Chem 77, 4726–4733 (2005). 16053282 10.1021/ac048303p

[b60] Unger M. A., Chou H. P., Thorsen T., Scherer A. & Quake S. R. Monolithic microfabricated valves and pumps by multilayer soft lithography. Science 288, 113–116 (2000). 10753110 10.1126/science.288.5463.113

[b61] Hwang Y. S. *et al.* Microwell-mediated control of embryoid body size regulates embryonic stem cell fate via differential expression of WNT5a and WNT11. Proc Natl Acad Sci USA 106, 16978–16983 (2009). 19805103 10.1073/pnas.0905550106 PMC2761314

[b62] Cavallini A., Baj-Rossi C., Ghoreishizadeh S., De Micheli G. & Carrara S. Design, fabrication, and test of a sensor array for perspective biosensing in chronic pathologies. Proc Int IEEE Conf BioCAS (2012).

[b63] Ges I. A. *et al.* Thin-film IrOx pH microelectrode for microfluidic-based microsystems. Biosensors Bioelectron 21, 248–256 (2005). 10.1016/j.bios.2004.09.021 16023951

[b64] Khademhosseini A. *et al.* Microfluidic patterning for fabrication of contractile cardiac organoids. Biomed Microdevices 9, 149–157 (2007). 17146728 10.1007/s10544-006-9013-7

