# Supplementary material for: Google Glass-Directed Monitoring and Control of Microfluidic Biosensors and Actuators
Source: Sci Rep. 2016 Mar 1;6:22237. doi: 10.1038/srep22237 (PMC4772091; doi:10.1038/srep22237)
Supplement: Supplementary Information [file srep22237-s1.pdf]

Supplementary Information

Google Glass-Directed Monitoring and Control of Microfluidic Biosensors and Actuators

Yu Shrike Zhang,[1,2,3] Fabio Busignani,[1,2,4,†] João Ribas,[1,2,5,6,†] Julio Aleman,[1,2,†] Talles Nascimento Rodrigues,[1,2,7] Seyed Ali Mousavi Shaegh,[1,2] Solange Massa,[1,2,8] Camilla Baj Rossi,[1,2,9] Irene Taurino,[1,2,9] Su-Ryon Shin,[1,2,3] Giovanni Calzone,[1,2,10] Givan Mark Amaratunga,[1,2,11] Douglas Leon Chambers,[1,2,12] Saman Jabari,[1,2,13] Yuxi Niu,[1,2,14] Vijayan Manoharan,[1,2] Mehmet Remzi Dokmeci,[1,2,3] Sandro Carrara,[9] Danilo Demarchi,[4] and Ali Khademhosseini[1,2,3,15,16,*]

[1]Biomaterials Innovation Research Center, Division of Biomedical Engineering, Department of Medicine, Brigham and Women's Hospital, Harvard Medical School, Cambridge, MA 02139, USA

[2]Harvard-MIT Division of Health Sciences and Technology, Massachusetts Institute of Technology, Cambridge, MA 02139, USA

[3]Wyss Institute for Biologically Inspired Engineering, Harvard University, Cambridge, MA 02139, USA

[4]Department of Electronics and Telecommunications, Politecnico di Torino, 10129 Torino, Italy

[5]Doctoral Programme in Experimental Biology and Biomedicine, Center for Neuroscience and Cell Biology, Institute for Interdisciplinary Research, University of Coimbra, 3030-789 Coimbra, Portugal

[6]Biocant — Biotechnology Innovation Center, 3060-197 Cantanhede, Portugal

[7]Federal University of São Francisco Valley, Centro, Petrolina, PE 56304-917, Brazil

[8]Programa de Doctorado en Biomedicina, Universidad de los Andes, Santiago 7620001, Chile

[9]Integrated Systems Laboratory, École Polytechnique Fédérale de Lausanne, 1015 Lausanne, Switzerland

[10]Department of Biomedical Engineering, Polytechnic University of Turin, Turin, Italy

[11]University of Pittsburgh, Pittsburgh, PA 15260, USA

[12]Department of Electrical Engineering and Computer Science, Massachusetts Institute of Technology, Cambridge, MA 02139, USA

[13]Hammond High School, Columbia, SC 29209, USA

[14]College of Life Sciences, Zhejiang University, Hangzhou 310027, China

[15]Department of Bioindustrial Technologies, College of Animal Bioscience and Technology, Konkuk University, Hwayang-dong, Gwangjin-gu, Seoul 143-701, Republic of Korea

[16]Department of Physics, King Abdulaziz University, Jeddah 21569, Saudi Arabia

[*]Author to whom any correspondence should be addressed. Email: alik@rics.bwh.harvard.edu

[†]These authors contributed equally to this work.

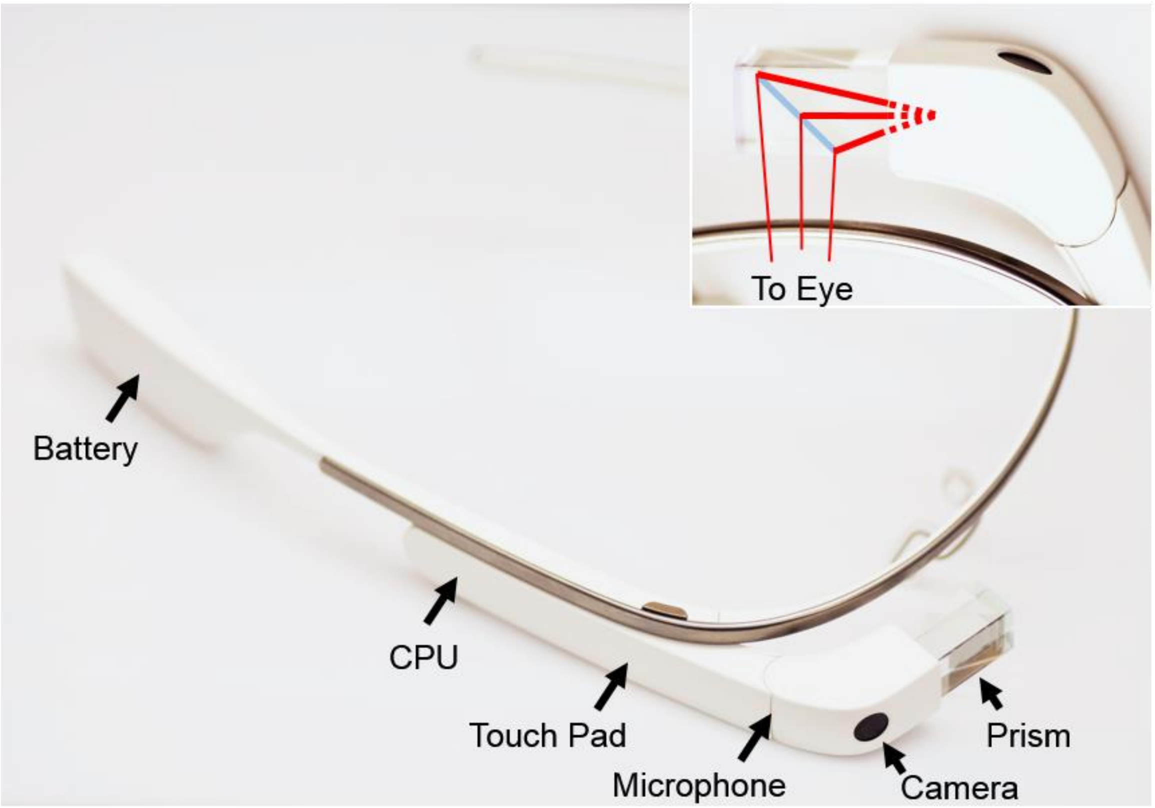

Supplementary Figure 1. Photograph and major components of the Google Glass.

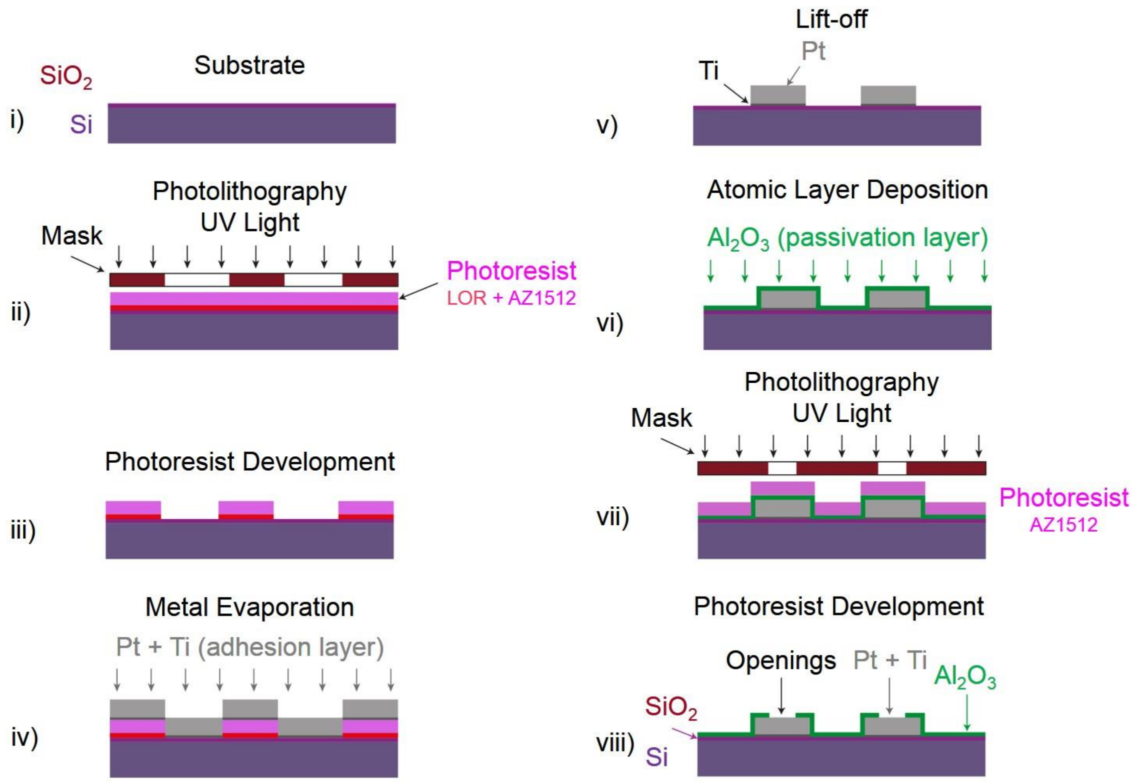

Supplementary Figure 2. Process flow to microfabricate the multi-sensor chip.

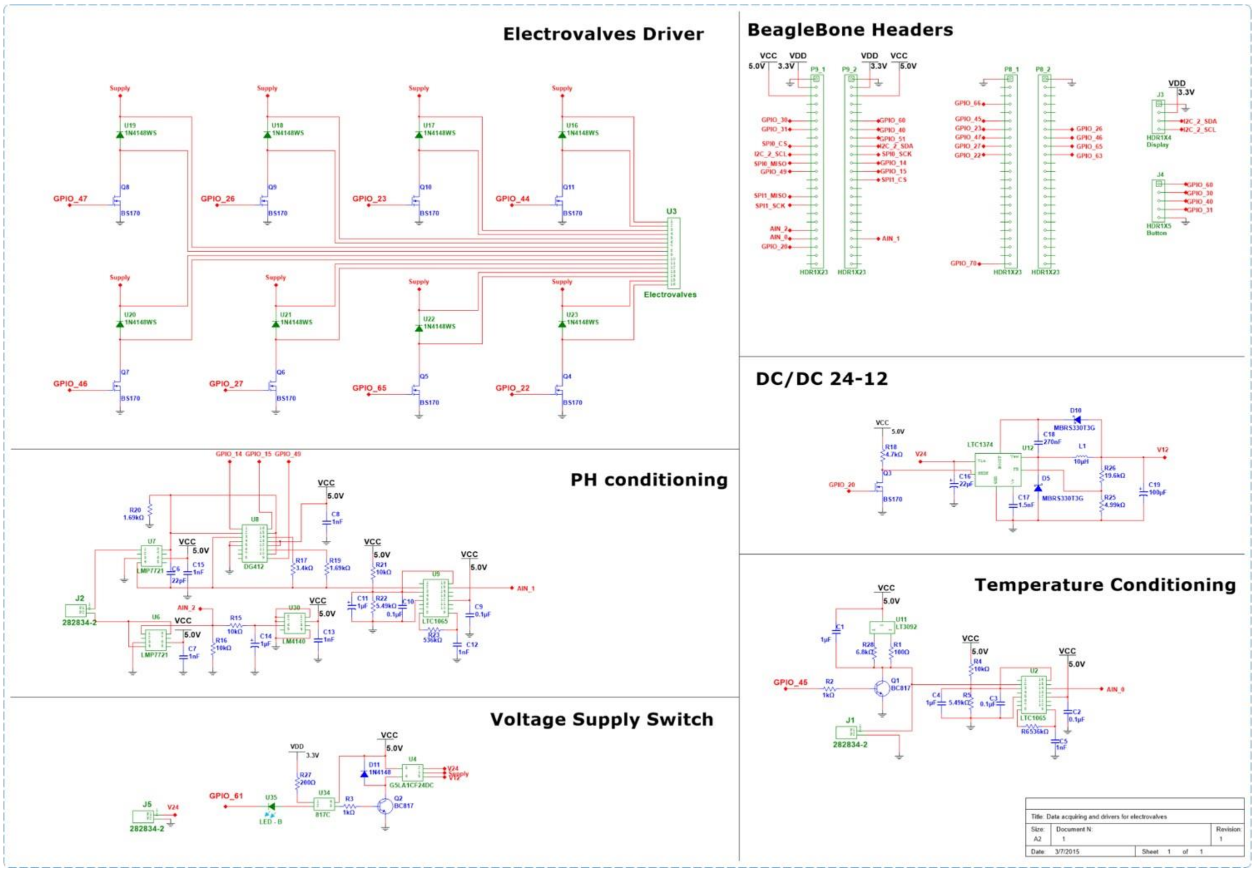

Supplementary Figure 3. Design of the electronic circuits for sensor data reading, electrovalve driver, and BeagleBone header.

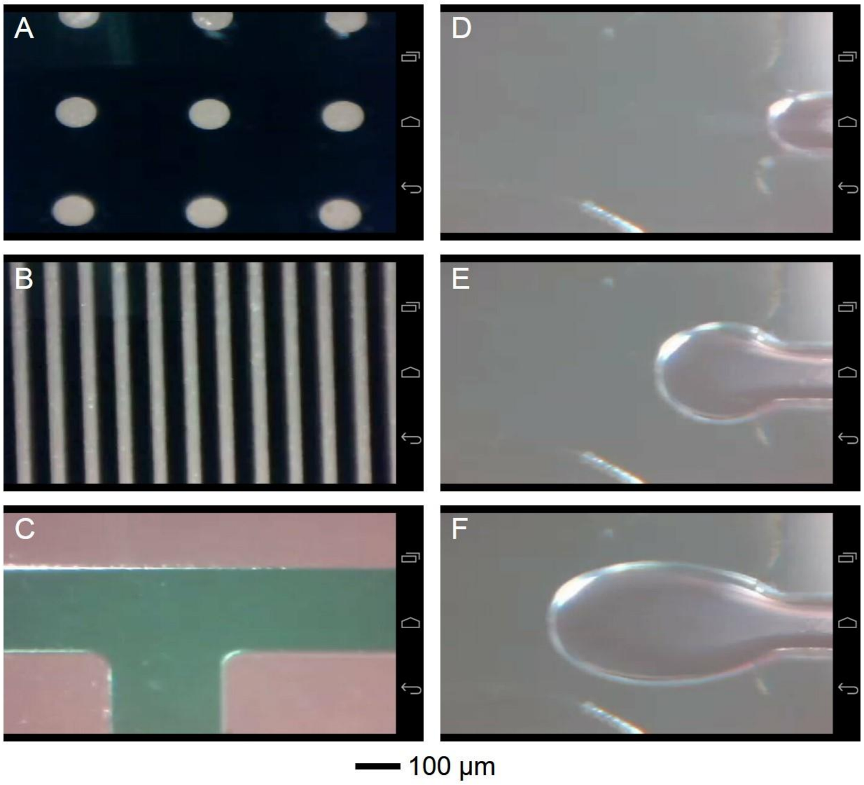

Supplementary Figure 4. Display of mini-microscopic observations on the Google Glass of A) a mask with dot arrays, B) a mask with line arrays, C) a T-shaped microfluidic channel filled with food dye, and E-F) sequential screenshots showing the formation of an aqueous droplet in oil in a microfluidic device.

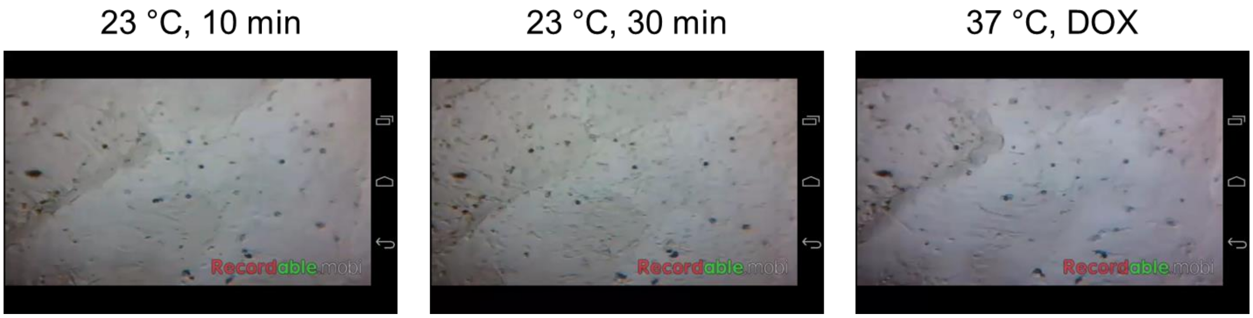

Supplementary Movie 1. Movie transmitted onto the Google Glass showing the beating of cardiomyocytes at 23 °C for 10 min after moving out from the incubator.

Supplementary Movie 2. Movie transmitted onto the Google Glass showing the beating of cardiomyocytes at 23 °C for 30 min after moving out from the incubator.

Supplementary Movie 3. Movie transmitted onto the Google Glass showing the beating of cardiomyocytes in the incubator at 37 °C post treatment with DOX.

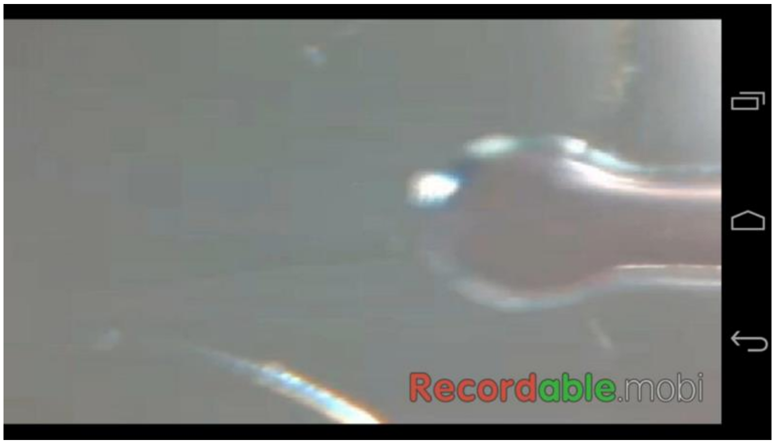

Supplementary Movie 4. Movie transmitted onto the Google Glass showing the mini-microscopic recording of the generation of aqueous droplets in oil using a microfluidic device.

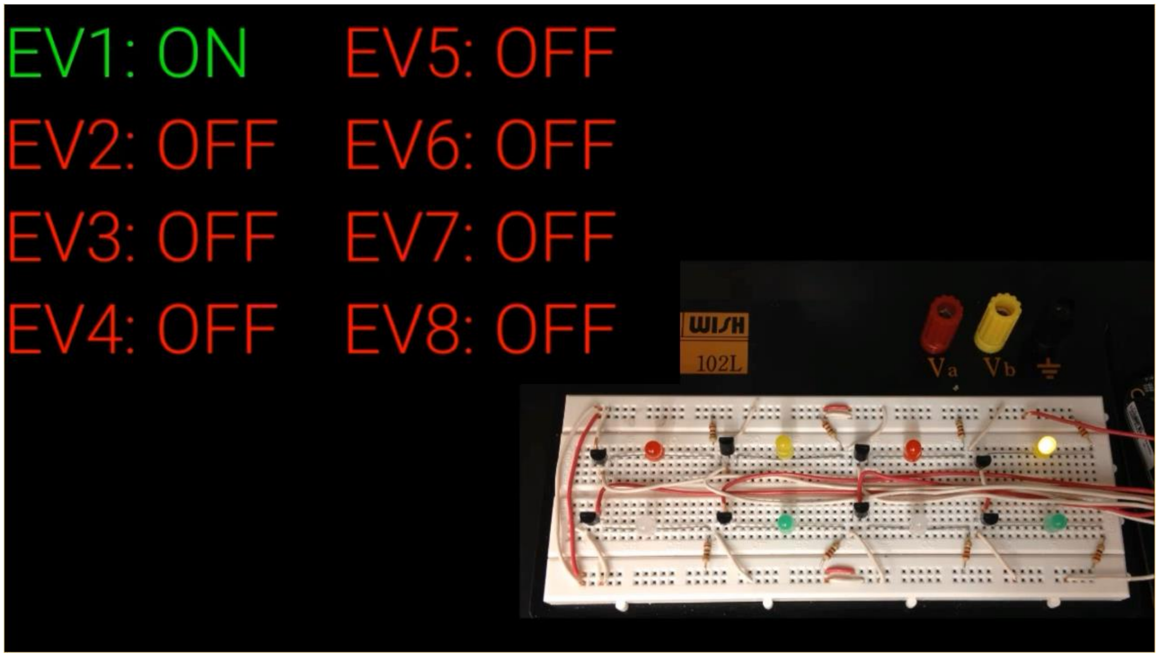

Supplementary Movie 5. Movie showing the control of LEDs from the Google Glass. LEDs were turned on and off one by one sequentially in order.

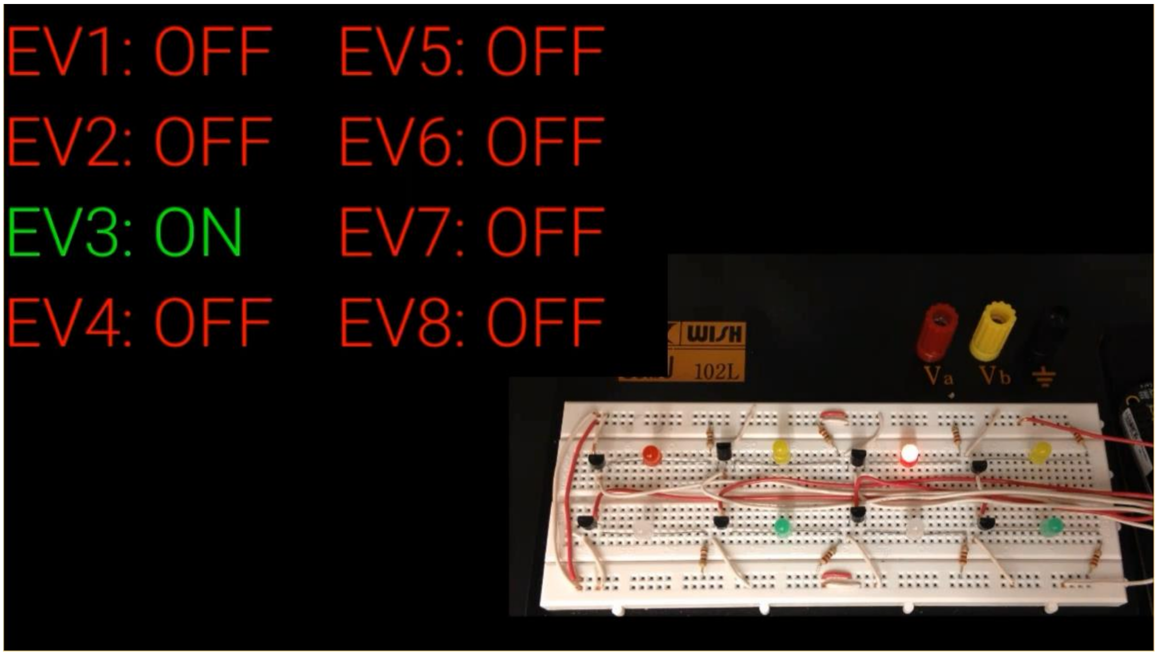

Supplementary Movie 6. Movie showing the control of LEDs from the Google Glass. LEDs were turned on and off one by one randomly.

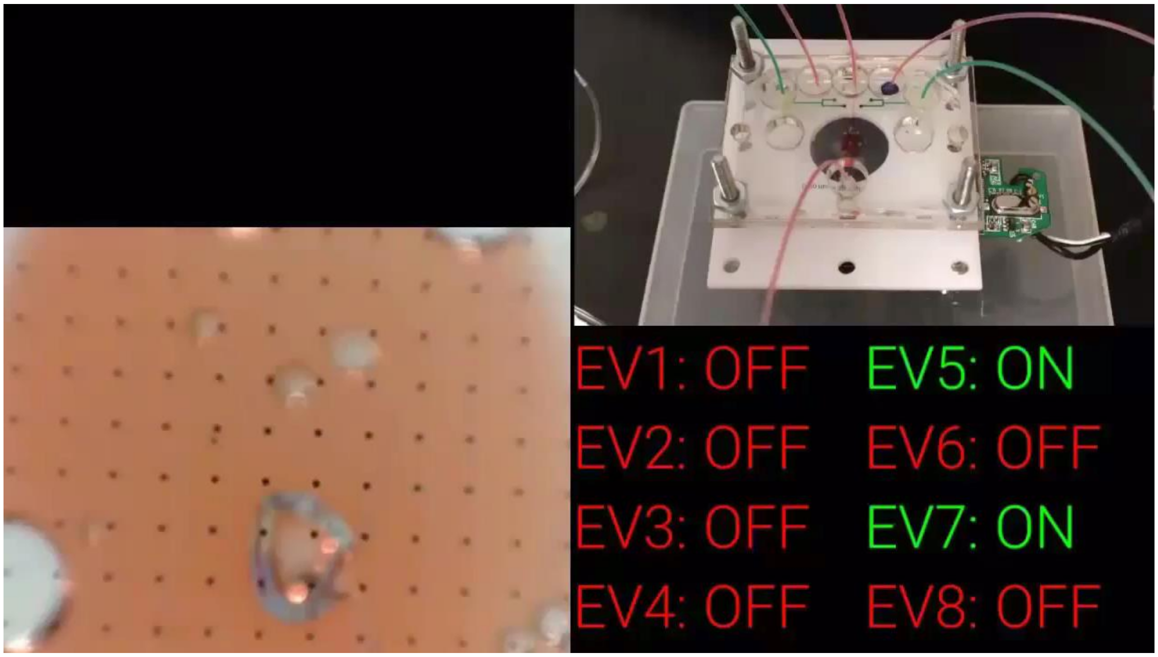

Supplementary Movie 7. Movie showing the control of electrovalves from the Google Glass for sequential activation/deactivation of multiple flow streams in to a microfluidic bioreactor.
